# Alterations of Pancreatic Islet Structure, Metabolism and Gene Expression in Diet-Induced Obese C57BL/6J Mice

**DOI:** 10.1371/journal.pone.0086815

**Published:** 2014-02-05

**Authors:** Regan Roat, Vandana Rao, Nicolai M. Doliba, Franz M. Matschinsky, John W. Tobias, Eden Garcia, Rexford S. Ahima, Yumi Imai

**Affiliations:** 1 Department of Medicine, Division of Endocrinology, Diabetes, and Metabolism, University of Pennsylvania Perelman School of Medicine, Philadelphia, Pennsylvania, United States of America; 2 Department of Biochemistry and Biophysics, University of Pennsylvania Perelman School of Medicine, Philadelphia, Pennsylvania, United States of America; 3 Bioinformatics Group, Penn Molecular Profiling Facility, University of Pennsylvania Perelman School of Medicine, Philadelphia, Pennsylvania, United States of America; 4 Department of Internal Medicine, Strelitz Diabetes Center, Eastern Virginia Medical School, Norfolk, Virginia, United States of America; University of Bremen, Germany

## Abstract

The reduction of functional β cell mass is a key feature of type 2 diabetes. Here, we studied metabolic functions and islet gene expression profiles of C57BL/6J mice with naturally occurring nicotinamide nucleotide transhydrogenase (NNT) deletion mutation, a widely used model of diet-induced obesity and diabetes. On high fat diet (HF), the mice developed obesity and hyperinsulinemia, while blood glucose levels were only mildly elevated indicating a substantial capacity to compensate for insulin resistance. The basal serum insulin levels were elevated in HF mice, but insulin secretion in response to glucose load was significantly blunted. Hyperinsulinemia in HF fed mice was associated with an increase in islet mass and size along with higher BrdU incorporation to β cells. The temporal profiles of glucose-stimulated insulin secretion (GSIS) of isolated islets were comparable in HF and normal chow fed mice. Islets isolated from HF fed mice had elevated basal oxygen consumption per islet but failed to increase oxygen consumption further in response to glucose or carbonyl cyanide-4-trifluoromethoxyphenylhydrazone (FCCP). To obtain an unbiased assessment of metabolic pathways in islets, we performed microarray analysis comparing gene expression in islets from HF to normal chow-fed mice. A few genes, for example, those genes involved in the protection against oxidative stress (hypoxia upregulated protein 1) and *Pgc1α* were up-regulated in HF islets. In contrast, several genes in extracellular matrix and other pathways were suppressed in HF islets. These results indicate that islets from C57BL/6J mice with NNT deletion mutation develop structural, metabolic and gene expression features consistent with compensation and decompensation in response to HF diet.

## Introduction

Insulin resistance commonly seen in obesity is considered a risk factor for the development of type 2 diabetes (T2D) [1]. However, the failure of pancreatic islet insulin secretion to compensate for insulin resistance is the critical pathology that ultimately leads to T2D [2]–[4]. The critical role islets play in the pathogenesis of T2D is evidenced by gene wide association studies (GWAS) that have identified susceptibility loci for T2D more frequently associated with islet functions than insulin sensitivity [5]. Moreover, the progressive worsening of T2D in humans is thought to result from a gradual loss of functional β cell mass [3]. Thus, there is strong interest in dissecting the molecular pathways that lead to the decline in mass and function of β cells in T2D, especially as the disease remains a serious public health challenge with limited numbers of effective therapies to reverse the pathology [6].

Various animal models of obesity and diabetes have been used to identify mechanisms responsible for the development of T2D, and to test the efficacy of therapeutic interventions [7]. C57BL/6J (BL6J) mouse on high fat diet (HF) has been one of the most commonly employed models due to its wide availability, and the ease of genetic manipulation [8]. In addition, the development of obesity in BL6J results from diet and multiple genetic susceptibility loci in BL6J, and thus mimics human obesity [9]. Of note, BL6J from the Jackson laboratories, widely used especially in the US, carries a naturally occurring deletion of functional nicotinamide nucleotide transhydrogenase (NNT) protein [10]. NNT, an antioxidant defense gene, catalyzes the production of NADPH that facilitates detoxification of reactive oxygen species (ROS) through the regeneration of reduced glutathione, and knockdown of NNT increases ROS. NNT mutation in BL6J is reported to reduce insulin secretion compared with BL6 without NNT mutation [10]. However, despite their wide use, the analyses that focus on functional, morphological, and gene expression changes in islets of this model with NNT mutation upon HF challenge are relatively limited. Considering the critical role of islets in the development and progression of T2D in humans, we aimed to obtain comprehensive metabolic and gene expression data in islets associated with diet-induced obesity in this T2D mouse model with NNT mutation. We have identified wide arrays of structural, secretory, metabolic, and gene expression alterations in islets from HF fed BL6J that implicate both adaptation and decompensation to insulin resistance.

## Materials and Methods

### Animal studies

Experiments were performed in accordance with the Institutional Animal Care and Use Committee guidelines with its approvals. 4 week-old male BL6J mice (Jackson Laboratories) were housed n = 5/cage in 12 hour light: dark cycle, at ambient temperature of 22°C, and allowed free access to food and water. Groups of mice were fed normal rodent chow (NC) (4 kcal% fat; 5001 from Lab Diet), or high fat diet (HF) (45 kcal% fat; D124551 from Research Diets, Inc.,). Mice were harvested for histological studies and for isolation of islets after 14 weeks on either NC or HF diet.

### *In vivo* glucose homeostasis

Body weight was monitored weekly in conscious mice on *ad libitum* feeding. Tail blood glucose was measured with a glucometer during daytime while *ad libitum* feeding (One Touch Ultra; Lifescan, Johnson & Johnson). Glucose tolerance tests were performed after overnight fast (16 hours). After measuring tail blood glucose, 1.5 gm/kg glucose solution was injected IP, and tail blood was drawn at various times. To assess glucose stimulated insulin secretion in vivo, mice were fasted for 5 hours in the morning, given 3 gm/kg glucose IP, and 20 µl of tail blood was obtained at the indicated times for insulin measurement by ELISA (Crystal Chem Inc.). Serum insulin levels were also measured in cardiac blood obtained at the time of sacrifice.

### Proliferation assay

Mice were continuously labeled with bromodeoxyuridine (5-bromo-2-deoxyuridine, BrdU) by providing 1 mg/ml of BrdU (Sigma-Aldrich) in the drinking water for 2 weeks [11]. Incorporation of BrdU into β cells was visualized by immunohistological analysis of harvested pancreas as described below.

### Histology

Pancreas from mice on regular rodent chow or high fat diet was weighed, fixed in 10% buffered formalin overnight, and embedded in paraffin. Morphometric analyses of pancreatic islets were performed [12]. Pancreatic sections were deparafinized, boiled six min in 10 mM citric acid (pH 6.0) for antigen retrieval, and incubated with 1∶400 guinea pig anti-insulin antibody at 4°C (Dako Diagnostics) overnight followed by 1∶400 biotinylated anti-guinea pig antibody at 37°C (Vector laboratories) for one hour. Color development was performed with DAB from Vector Laboratories according to the manufacture's protocol. Images of a pancreatic section cut through the maximum footprint of pancreas from each mouse were captured by a color video camera attached to a Nikon light microscope, and a composite picture covering a entire section was obtained. Then, border of a pancreatic section was visually defined on a composite picture of entire section displayed on a computer screen. Thereafter, pancreas area, β cell area, and islet size were measured using IP lab program (Scanalytics). The β cell area (%) was calculated as (sum of β cell area/total pancreas area). β cell mass was calculated as (weight of prefixed pancreas) × (β-cell area/total pancreas area). For BrdU visualization, pancreatic secretions were incubated with primary antibodies after antigen retrieval overnight at 4°C and visualized by Cy2 (for anti BrdU antibody) or Cy3 (for guinea pig anti-insulin antibody) conjugated secondary antibodies (1: 600, Jackson Immuno Research) at room temperature for two hours. Sheep anti BrdU antibody was used at 1∶1000 (US Biological). 4′, 6-diamidino-2-phenylindole (DAPI) was used for nuclear staining.

### Islet isolation and *ex vivo* perifusion assay

Mice were anesthetized with sodium pentobarbital (50 mg/kg i.p.), and pancreatic islets were isolated using collagenase digestion followed by Ficoll density gradient centrifugation as was described before [13]. Around 100 freshly isolated islets were loaded to a perifusion apparatus and perifused for 35 min with the Krebs buffer (pH 7.4) containing 2.2 mM Ca^2+^, 0.25% bovine serum albumin (BSA), 10 mM HEPES, and 3 mM glucose under 5% CO_2_ atmosphere at 37°C, followed by the Krebs buffer with 30 mM glucose for 20 min. At the end of each experiment, islets were tested for maximum insulin secretion by adding 30 mM KCl to the perifusate. Samples were collected at 1 ml/min for insulin measurement by radioimmunoassay (Linco research, Inc.) [13].

### Oxygen consumption

Oxygen consumption (VO_2_) was measured in 200 of isolated islets with a Clark electrode (model 949, Strathkelvin Instruments) in a water-jacked glass reaction vessel (Mitocell MT200) holding 175 µl of stirred, air-saturated modified Hank's buffer at 37°C as described before [14], [15]. The concentration of oxygen in the buffer was equal to 217 nmol/ml, as calculated from air solubility in water and the percentage of oxygen in dissolved air. The electrode signal was amplified and differentiated electronically (Oxygen Metter Model 781). Pancreatic islets isolated as above were pre-incubated in the Hank's buffer without glucose for 30 min. 200 islets were then loaded to the vessel. The basal oxygen consumption was first recorded for 3 min. Thereafter, 5 µl of buffer with glucose was injected into the vessel through a capillary in the ground-glass stopper to establish final concentration of 25 mM for the second phase of respirometry (about 3–5 min). Carbonyl cyanide-4-trifluoromethoxyphenylhydrazone (FCCP, final concentration 5 μM), which serves as a test of the degree of maximum efficiency of oxidative phosphorylation, was finally added to the vessel in a third phase of the test. The oxygen consumption after glucose loading was recorded for 15 min. Concentration of FCCP was chosen to obtain maximum respiratory capacity.

### RNA extraction and gene expression analyses

RNA was extracted from freshly isolated islets using RNeasy kit (Qiagen), and cDNA was generated by SprintPowerScript for cDNA synthesis (Clontech) using 500 ng of islet RNA as a template. Gene expression was analyzed with the ABI Prism 7900HT sequence detection system (Applied Biosystems) with commercial primers for the system. The results were expressed using 36B4 gene expression as an internal standard.

### Microarray analyses

Microarray analyses were performed on quadruplicate RNA samples of pancreatic islets from BL6J mice on NC or HF diet. Pancreatic islets from two mice were combined to yield one sample of islet RNA. All protocols were conducted as described in the Affymetrix GeneChips Expression Analysis Technical Manual (Affymetrix) using 5 µg total RNA and GeneChip Mouse Expression Arrays MOE 430v2 (Affymetrix). For data analyses, probe intensity data (cel files) were input into ArrayAssist Lite version 3.4 (Stratagene), and expression values for the probe sets were calculated using GCRMA. Affymetrix “present”, “absent” or “marginal” flags were also calculated. Subsequently intensity and flag data were imported into GeneSpring GX version 7.3.1 (Agilent Technologies) and filtered to retain probe sets flagged as “present” in at least three out of eight samples. Finally, the statistical test Significance Analysis of Microarrays (SAM, v2.23b, Stanford University) was applied using a two-class unpaired analysis, and differentially expressed genes were identified using a fold change cutoff of ≥1.5 (up or down) and a false discovery rate of 0.13%. The genes identified as down-regulated in HF group that show the reduction below 0.5 (239 genes) were uploaded to DAVID (http://david.abcc.ncifcrf.gov) and functional annotation chart were obtained. The genes “present” in at least 3 samples out of 8 were used as the background list. Microarray data were submitted to Gene expression omnibus (www.ncbi.nlm.nih.gov/projects/geo) under the accession number GSE23325.

### Statistics

The data are presented as mean ± SEM. Differences between two groups were assessed with a repeated measure ANOVA, or unpaired Student's *t* test. *p*<0.05 was considered significant.

## Results

### (i) BL6J mice partially compensate for insulin resistance on high fat diet

**Figure 1:**
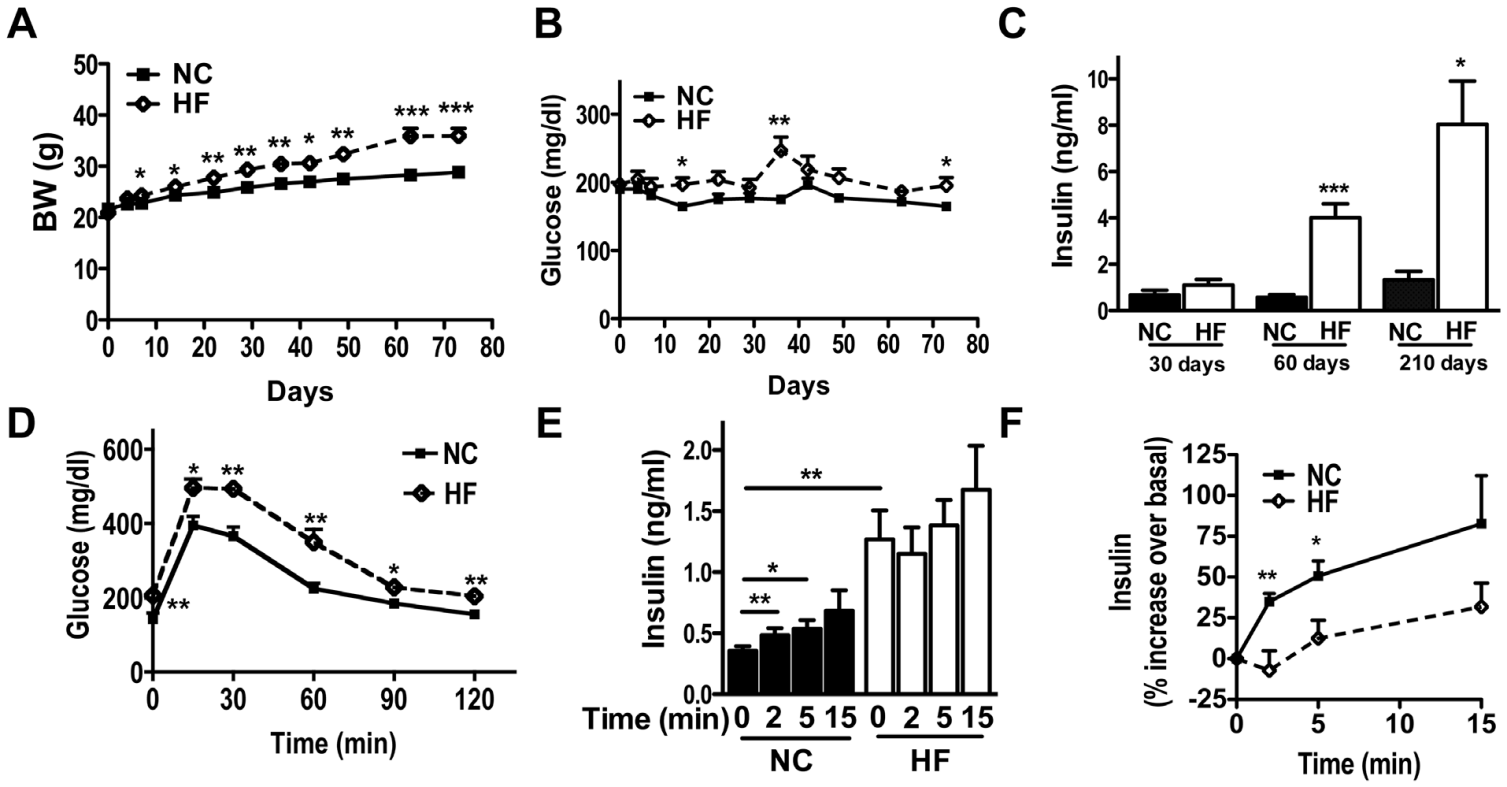
In vivo glucose homeostasis and insulin secretion in BL6J on high fat diet. (A–C) Male Bl6J mice were weaned to regular rodent chow (NC, 4.5% kcal fat diet) or high fat diet (HF, 45% kcal fat diet) and allowed free access to food. Body weight (BW, A), tail blood glucose (B), and blood insulin levels (C) were determined at indicated time. (D–F) I.P. Glucose Tolerance Test was performed in Bl6J mice on NC or HF diet for 3 months. (D) Blood glucose levels and (E) insulin levels during the test. (F) The increase in serum insulin levels was expressed taking the value at the time 0 as 100%. Data are mean ± s.e.m., (A–B) both n = 7, a repeated measures ANOVA *p*<0.005. (C) n = 10–14. (D) n = 7, a repeated measures ANOVA *p*<0.005. (E) n = 4–5, a repeated measures ANOVA *p*<0.05. (F) n = 4–5. * *p*<0.05, ** *p*<0.01, *** *p*<0.005 vs. control.

We determined whether BL6J developed a progressive decline in glucose homeostasis that is commonly seen in human T2D [3]. Male BL6J mice were fed a regular rodent chow (4 kcal% fat, NC) or high fat diet (45 kcal% fat, HF) at the time of weaning, the protocol that induces insulin resistance [16], and were monitored for changes in glucose homeostasis over time. As is shown in Fig. 1A, the body weight increased steadily in male BL6J mice on HF compared with NC (*p*<0.005, a repeated measures ANOVA). Blood glucose levels during daytime while *ad libitum* feeding increased significantly in HF by 14 days (*p*<0.05, Fig. 1B). The difference in glucose levels between 2 groups progressively increased up to 35 days, somewhat reduced to 63 days, and increased again at 70 days on diet, implicating complex interactions between blood glucose homeostasis, age, and diets (Fig. 1B). Serum insulin levels were higher in HF than NC, indicating that this mouse model is able to compensate for insulin resistance after prolonged HF treatment (Fig. 1C). Next we assessed *in vivo* insulin secretion in response to an intraperitoneal (IP) glucose load after 3 months of HF. Blood glucose levels were higher in HF mice compared with NC (Fig. 1D, a repeated measures ANOVA *p*<0.05). Although the insulin levels were higher at time 0 in HF group, they did not increase significantly in response to glucose. In contrast, insulin levels increased significantly in NC group (Fig. 1E). Also the percent increase in glucose stimulated insulin secretion (GSIS) was blunted in HF mice (Fig. 1F, a repeated measures ANOVA *p*<0.05). These in vivo results implicate that HF islets may compensate for insulin resistance (i.e. progressive hyperinsulinemia), but incompletely (i.e. blunted increase of insulin secretion upon a glucose load).

### (ii) Pancreatic islets from BL6J mice on high fat diet are larger but abnormal

**Figure 2:**
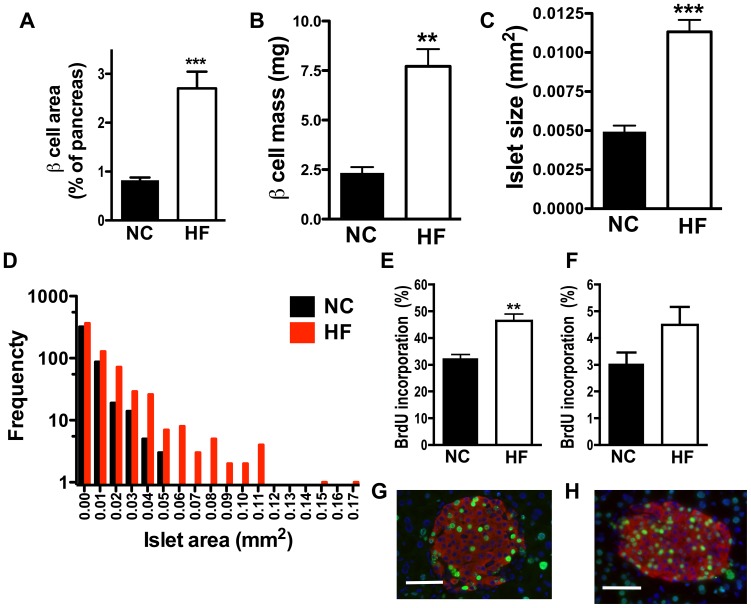
Morphometric analysis assessed islets from HF fed BL6J. (A) Total β cell area per pancreas, (B) β cell mass, and (C) average area of an individual islet in pancreatic sections from mice on normal rodent chow (NC) and high fat (HF) diet were compared The distribution of area of an individual islet is shown in (D). 6 sections for NC and 5 sections for HF fed mice were analyzed for (A–B). 4 sections per group from NC and HF fed mice with total of 448 islets for NC and 653 islets for HF were analyzed for (C–D). (E–H) The percentage of β cells positive for anti BrdU antibody was compared between NC and HF fed mice labeled with BrdU at 1 month of age (E), and 4 month of age (F). 4 sections per group were analyzed for (E) and 5 sections per group were analyzed for (F). Representative photos from an islet labeled at 1 month of age on NC (G) and HF (H) are shown. Green staining: BrdU. Red staining: insulin. Scale bars indicate 50 µm. Data are mean ± s.e.m., n = 4–5 for (E) and (F). ** *p*<0.01 vs. NC, *** *p*<0.005 vs. NC.

Since the in vivo studies suggested that HF islets display incomplete islet compensation, we compared islet morphology and secretory function of HF and NC islets. Morphometric analysis confirmed a significant increase in the total β cell area and β cell mass in HF islets as we reported before (Fig. 2A–B) [12]. The islet size expressed as area of an individual islet was also greater in HF than NC mice (Fig. 2C). The distribution of size of an individual islet showed that medium size of islet is also shifted to right for HF islets (0.00115 for NC vs. 0.00346 for HF, Fig. 2D). To determine the basis for larger islet size in HF mice, we administered BrdU in the drinking water during the first 2 weeks (Figs. 2E, G, H) or the 12^th^ through the 14^th^ week on the HF diet (Fig. 2F), and analyzed BrdU incorporation into β cells. As is shown in Figs. 2E–F, total incorporation of BrdU into β cells in 1 month old mice was 10 times higher than in 4 month old mice on both HF and NC diets, confirming an active proliferation of β cells in the younger mice [17]. BrdU incorporation into HF β cells was 1.43 times greater than NC (*p<0.01*) when labeled at an earlier age (Fig. 2E). There was no difference in BrdU incorporation into β cells at an older age between the two groups (Fig. 2F), indicating that β cell proliferation has limited role in compensation to HF diet at an older age.

**Figure 3:**
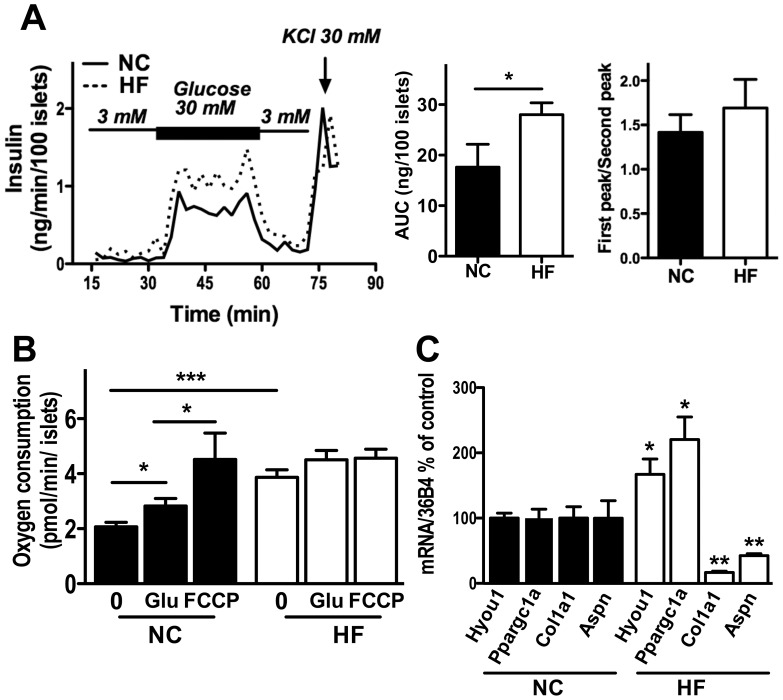
Changes in secretory and metabolic functions, and gene expression in islets from HF fed BL6J. (A) Glucose–stimulated insulin secretion (GSIS) was compared ex vivo between islets from mice on NC versus HF diets. Profiles of insulin secretion, area under the curve (AUC) of insulin secretion, and the ratio of first and second peak of insulin secretion are shown. (B) Oxygen consumption was compared between islets from NC and HF incubated without glucose (0), followed by treatment with 25 mM glucose (Glu), and then with carbonyl cyanide-4-trifluoromethoxyphenylhydrazone (FCCP). (C) rtPCR compared hypoxia up-regulated protein 1 (*Hyou1*), peroxisome proliferator-activated receptor gamma coactivator 1-alpha, (*Ppargc1a*), collagen, type I, alpha 1, (*Col1a1*), and asporin (*Aspn*) expression between islets from NC and HF groups. The results were expressed using 36B4 gene expression as an internal. Data are mean ± s.e.m., n = 4–5. * *p*<0.05, ** *p*<0.01, *** *p*<0.005 vs. control.

We have previously shown that islets from HF BL6J have increased insulin secretion ex vivo when evaluated with a glucose ramp from 0 to 30 mM [12]. We tested whether islets from HF mice show a defect in insulin secretion in response to an acute increase of glucose, which is characteristic of the defect in first phase insulin secretion seen in human T2D [18]. Ex vivo perifusion showed that an acute rise of glucose from 3 mM to 30 mM increased insulin secretion in islets from both NC and HF mice in a similar fashion (Fig. 3A). A delayed peak insulin secretion after sustained exposure to 30 mM glucose was also seen in both NC and HF islets. Therefore, there was no obvious disruption of either first phase or second phase insulin secretion from HF islets ex vivo (Fig. 3A). Although, repeated measure ANOVAs did not reach statistical significance, area under the curve (AUC) of insulin secretion per islet was increased in HF islets (*p*<0.05). Next, we compared VO_2_ in HF and NC islets. The oxygen consumption rate is an important manifestation of stimulus-secretion coupling and serves as the index of efficiency of oxidative phosphorylation [14]. VO_2_ at baseline (0 mM glucose) was 1.87 times greater in HF compared with NC islets when expressed per islet (Fig. 3B, *p*<0.005), which likely reflects larger size of individual islets in HF fed mice. In addition, the increment in VO_2_ in response to glucose was blunted in HF islets (Fig. 3B). Incubation with 30 mM glucose increased VO_2_ of NC islets to 1.35 times the basal level (*p*<0.05), while glucose treatment did not change VO_2_ significantly in HF islets (Fig. 3B). Moreover, treatment with FCCP, an agent that induces maximal oxidative phosphorylation, failed to increase VO_2_ in HF islets, while increasing VO_2_ in NC islets 2.18 times the baseline (Fig. 3B). This implies the alteration in oxidative phosphorylation process of HF fed islets despite of seemingly unaltered insulin secretion from HF fed islets in perifusion assay (Fig 3A).

### (iii) Pancreatic islet gene expression in HF versus NC mice

To determine the effects of HF diet on islet gene expression profile in an unbiased manner, microarray compared islets from NC and HF fed mice. Out of 25,617 probes tested, we observed up-regulation of 44 probe sets representing 34 genes and down-regulation of 509 probe sets representing 391 genes in HF islets using a fold change cutoff of ≥1.5 and a false discovery rate of ≥0.13% (Fig. 3C, Table S1 for up-regulation and Table S2 for down-regulation). With our stringent criteria there were a limited number of genes that were increased in HF islets. One of the up-regulated genes, *Hyou1*, is an ER chaperon protein that is considered to be protective against ER stress and previously shown to be increased in human T2D islets compared with non-diabetic islets [19]. rtPCR confirmed that *Hyou1* was increased by 1.7 fold in HF vs. NC islets (Fig. 3C). *Pgc1a* was identified by microarray and validated by rtPCR to be increased to 2.2 fold in HF vs. NC islets (Fig. 3C). Previously, *Pgc1α* was shown to be up-regulated in other rodent models of islet hypertrophy and hyperglycemia including *ob/ob* mice and ZDF mice [20]. In contrast to the limited number of genes whose expression was increased in HF islets, there were 12 times more genes that were down-regulated in HF islets compared with control. To elucidate functional features of these down-regulated genes, functional Annotation Clustering was performed using DAVID Bioinformatics Resources (http://david.abcc.ncifcrf.gov) for genes whose expression was reduced below 0.5 of control. The analyses showed that genes involved in extracellular matrix (*p* = 1.6 E-31, GOTERM_CC_FAT, enrichment compared with background genes), and those involved in immune response (*p* = 8.0 E-6 GOTERM_BP_FAT, enrichment compared with background genes) were reduced in HF islets. The reduction of two genes from extracellular matrix cluster was validated by rtPCR. Expression of *Col1a1*, the major component of type 1 collagen was reduced to 0.17 in HF islets. Similarly, *asporin*, an extracellular matrix protein that belongs to a family of small leucine-rich repeat proteoglycan, was also reduced to 0.43 of the levels in NC (Fig. 3C). These results implicate the structural change of HF islets compared with NC islets.

## Discussion

The BL6J inbred mouse from Jackson laboratories has been widely used as a model of obesity and diabetes due to their susceptibility for obesity on HF feeding [9]. Numerous studies have assessed the contribution of genes or therapeutics to islet health and functions in this BL6J on HF. The objective of the current study was to obtain comprehensive assessments of structural, metabolic and gene expression in HF islets. There have been studies that focused on the effects of HF diet on islet morphology and insulin secretion in various stains of mice [21]–[23]. However, it is noteworthy that few studies have examined the alterations of islet mass and function simultaneously in this HF-fed BL6J sub-strain of mice despite of wide use. Our results demonstrate that HF islets of male BL6J show a combination of structural and secretory compensation for insulin resistance, as well as metabolic dysregulation.

It has been reported that BL6J strain from the Jackson laboratories carries a naturally occurring NNT mutation that impairs insulin secretion [10]. However, our study showed that the same BL6J strain was still capable of increasing insulin secretion in response to HF diet. Mild hyperglycemia and persistently elevated serum insulin levels were observed in HF male mice in the current study, similar to female BL6J mice from Taconic (Denmark) placed on 58% kcal fat diet, which do not harbor the NNT mutation [21]. C57Bl/6N from Charles River Laboratories in Japan did not show hyperglycemia after 18 weeks of 52% kcal fat diet [24]. Thus, some of the compensatory mechanisms for islet adaptation to insulin resistance are not impaired by NNT mutation in BL6J. The mild hyperglycemia in BL6J on HF diet differs from the natural history of T2D in humans, in which hyperglycemia tends to worsen with time [3]. The progressive hyperinsulinemia is associated with the increase in islet mass in the current (BL6J with NNT mutation) and previous study (Bl6J with NNT mutation) [25], [26]. On the other hand, human T2D is characterized by both loss of islet mass and function [3], [18]. Prevention of islet mass expansion due to aging or autophagy defects exacerbates hyperglycemia in HF mice [25], [27]. Thus, it is possible that the compensatory increase in islet mass plays a critical role in preventing the worsening of hyperglycemia in HF BL6J mice. Islet enlargement may be explained, at least partly, by increased proliferation as was indicated by higher BrdU incorporation in HF compared to NC islets. Likewise, BL6J mice without NNT mutation also showed an increase in proliferation on HF based on Ki67 staining [23]. A BL6J x DBA/2J hybrid mouse fed 45% fat diet for 1 year from age 7 to 8 week showed an increase in β cell number, indicating that proliferation contributes to enlargement of HF islets in mice from various genetic backgrounds [22]. In addition, a reduction in apoptosis may have contributed to a higher β cell number in HF islets in C57BL/6 mice obtained from Taconic [26].

Mice on HF displayed baseline hyperinsulinemia and a blunted in vivo glucose-stimulated insulin secretory response. On the other hand, HF islets did not show a clear alteration in temporal profiles of insulin secretion when challenged with glucose ex vivo. The ex vivo perifusion was performed with freshly isolated islets, after islets had been pre-incubated in low glucose buffer (54 mg/dl) before applying the glucose ramp. This procedure may have elicited a more robust increase in insulin secretion in comparison to in vivo experiments where islets were exposed to elevated levels of glucose at a baseline (mean glucose levels at 206.6 mg/dl for HF and at 142 mg/dl for NC mice, Fig. 1D). Interestingly, HF islets in the current study showed the blunting of the VO_2_ increase after pharmacologically induced maximal oxidative phosphorylation, suggesting an alteration of this process in mitochondria of HF fed islets in the basal state. Other functional changes previously described in HF islets include impairments in voltage gated calcium influx, exocytosis, glucose induced increase in ATP, and glucose induced change in Ca^2+^ [23], [28]. These abnormalities may impair the glucose responsiveness of β cells when they are uninterruptedly exposed to hyperglycemic conditions in vivo (Fig. 1E). Alternatively, neurological and paracrine factors may play a role in the blunting of glucose stimulated insulin secretion in vivo.

Our microarray analysis identified *Pgc1α* as one of the genes up-regulated in HF islets. The expression of *Pgc1α* in islets was also reported to be elevated in several animal models that have increased demand for insulin secretion such as *ob/ob* mice, mice after partial pancreatectomy, and Zucker Diabetic Fatty (ZDF) rats. However, the regulation of PGC1α expression and its function in islets appears complex. The same study proposed that PGC1α in the islets negatively regulated insulin secretion, since forced expression of *Pgc1α* in islets reduced insulin secretion [20]. At the same time, *PGC1α* expression was reported to correlate with higher insulin secretion in human islets, streptozotocin treated mice, and Goto-Kakizaki (GK) rats [29]. Since the reduction of *PGC1α* by siRNA in dispersed human islets reduced insulin secretion, the maintenance of basal levels of PGC1α may be necessary to support insulin secretion [29].

*Hyou1* is another gene that is increased in HF islets in our microarray analysis. HYOU1 is an ER chaperon protein whose expression increases in various tissues under hypoxia, but is highly expressed in the liver and pancreatic β cells even at normoxia [30], [31]. Higher expression of *Hyou1* is considered to be protective against ER stress and shown to reduce insulin resistance when systemically overexpressed in mice [32], [33]. Interestingly the amelioration of lipid-induced ER stress in the liver by Sirt1/AMPK pathway activation is associated with induction of HYOU1 [34]. There are several studies demonstrating potential importance of HYOU1 in islet functions. Proteomic study previously showed that HYOU1 is increased in human T2D islets compared with non-diabetic islets [19]. Moreover the reduction of *Hyou1* decreased glucose stimulated insulin secretion in MIN6 cells, supporting its critical role in insulin secretion [35]. Thus, the increased expression of *Hyou1* in pancreatic islets may serve as a protective mechanism in BL6J on HF diet.

Our microarray study revealed a reduction of ECM genes in islets of HF fed mice. This included the decrease in *Col1a1* and *asporin*, implicating the reduction in tissue fibrosis in HF islets [36]. COl1A1 is the major component of type 1 collagen that is the most abundant collagen in connective tissues [36]. Asporin is an extracellular matrix protein that is proposed to play a role in tissue remodeling and interact with TGF- β1 and BMP-2 [37], [38]. In contrast, an increase in fibrosis and reduced islet mass have been reported in several rat models of T2D including ZDF rat, GK rat, Otsuka Long-Evans Tokushima fatty rats, and spontaneously diabetic Torii rat [39]–[42]. Of interest, the microarray study of the GK rats revealed an up-regulation of multiple genes involved in ECM, in agreement with histological study [39]. Although the excess accumulation of ECM indicates fibrosis and likely negatively correlates with islet function, it is also widely accepted that ECM is supportive for islet function [43]. Interestingly, we previously demonstrated that AKR/J, an inbred mouse strain that compensates for HF diet better than BL6J mice, has greater expression of ECM [13]. Further studies are required to determine whether the reduction of ECM genes in islets of BL6J mice on HF diet is associated with islet compensation or functional alteration.

In summary, we performed morphological, secretory, metabolic, and gene expression profiling studies to characterize the changes in islets of BL6J with NNT mutation on HF diet. We observed features demonstrating a combination of islet compensation and functional impairment in this widely used model of T2D. The information gained from our study will aid the interpretation of studies that utilize BL6J with NNT mutation as a model of diet induced obesity, and facilitate the translation of these data to human T2D investigation.

## Supporting Information

Table S1 The list of genes up-regulated in HF islets compared with NC islets in the microarray analysis using a fold change cutoff of ≥1.5 and a false discovery rate of ≥0.13%.
(DOC)

Table S2 The list of genes down-regulated in HF islets compared with NC islets in the microarray analysis using a fold change cutoff of ≥1.5 and a false discovery rate of ≥0.13%.
(DOC)
