# Supplementary material for: Alterations of Pancreatic Islet Structure, Metabolism and Gene Expression in Diet-Induced Obese C57BL/6J Mice
Source: PLoS One. 2014 Feb 5;9(2):e86815. doi: 10.1371/journal.pone.0086815 (PMC3914796; doi:10.1371/journal.pone.0086815)
Supplement: Table S1 — The list of genes up-regulated in HF islets compared with NC islets in the microarray analysis using a fold change cutoff of ≥1.5 and a false discovery rate of ≥0.13%. (DOC) [file pone.0086815.s001.doc]

Table S1

|  | Fold Change | Affimetrix ID | REFSEQ_mRNA |
| --- | --- | --- | --- |
| **Receptor/ion channel & transporter** |  |  |  |
| GAMMA-AMINOBUTYRIC ACID (GABA-A) RECEPTOR, SUBUNIT ALPHA 4 | 5.84 | 1433707_AT | NM_010251 |
| TUMOR NECROSIS FACTOR RECEPTOR SUPERFAMILY, MEMBER 23 | 4.08 | 1422101_AT | NM_024290 |
| GUANYLATE CYCLASE 2C | 3.18 | 1436370_AT | NM_145067 |
| TRANSIENT RECEPTOR POTENTIAL CATION CHANNEL, SUBFAMILY C, MEMBER 4 | 2.24 | 1451033_A_AT | NM_016984 |
| GROUP SPECIFIC COMPONENT | 2.12 | 1426547_AT | NM_008096 |
| POTASSIUM VOLTAGE-GATED CHANNEL, SHAKER-RELATED SUBFAMILY, BETA MEMBER 3 | 1.73 | 1421790_A_AT | NM_010599 |
| SOLUTE CARRIER FAMILY 4, SODIUM BICARBONATE COTRANSPORTER-LIKE, MEMBER 10 | 1.58 | 1417672_AT | NM_033552 |
| ATPASE, CA++ TRANSPORTING, UBIQUITOUS | 1.52 | 1450124_A_AT | NM_016745 |
|  |  |  |  |
| **Lipid Metabolism** |  |  |  |
| PEROXISOME PROLIFERATIVE ACTIVATED RECEPTOR, GAMMA, COACTIVATOR 1 ALPHA | 4.96, 3.20 | 1460336_AT, 1437751_AT | NM_008904 |
| APOLIPOPROTEIN F | 1.78 | 1418239_AT | NM_133997 |
| FATTY ACID BINDING PROTEIN 3, MUSCLE AND HEART | 1.77 | 1416023_AT | NM_010174 |
| PHOSPHATIDYLINOSITOL 3-KINASE, REGULATORY SUBUNIT, POLYPEPTIDE 1 (P85 ALPHA) | 1.68 | 1425514_AT | NM_001024955, NM_011085 |
| OXYSTEROL BINDING PROTEIN-LIKE 3 | 1.51 | 1428484_AT | NM_027881 |
|  |  |  |  |
| **Oxidative stress/hypoxia** |  |  |  |
| HYPOXIA UP-REGULATED 1 | 3.86 | 1451913_A_AT | NM_021395 |
| GLUTATHIONE PEROXIDASE 2 | 1.92 | 1449279_AT | NM_030677 |
|  |  |  |  |
| **Transcription Regulator** |  |  |  |
| PEROXISOME PROLIFERATOR ACTIVATED RECEPTOR BINDING PROTEIN | 1.89 | 1421907_AT | NM_013634, NM_134027 |
| CYSTEINE-RICH HYDROPHOBIC DOMAIN 1 | 1.61 | 1427573_AT | NM_009767 |
| LEUCINE ZIPPER TRANSCRIPTION REGULATOR 2 | 1.61 | 1450734_AT | NM_033354 |
| HOMEODOMAIN INTERACTING PROTEIN KINASE 3 | 1.55 | 1419191_AT | NM_010434 |
| CREBBP/EP300 INHIBITORY PROTEIN 1 | 1.52 | 1448406_AT | NM_025613 |
|  |  |  |  |
| **Small GTP binding proteins** |  |  |  |
| RASD FAMILY, MEMBER 2 | 15.53 | 1427344_S_AT | NM_029182 |
| RAB39B, MEMBER RAS ONCOGENE FAMILY | 1.90 | 1453246_AT | NM_175122 |
| RAB3D, MEMBER RAS ONCOGENE FAMILY | 1.58 | 1418890_A_AT | NM_031874 |
|  |  |  |  |
| **Glycoprotein/proteoglycan** |  |  |  |
| SYNDECAN 4 | 2.01 | 1448793_A_AT | NM_011521 |
| MUCIN 4 | 1.79 | 1438555_X_AT | NM_080457 |
| HYALURONAN AND PROTEOGLYCAN LINK PROTEIN 4 | 1.53 | 1435650_AT | NM_177900 |
|  |  |  |  |
| **Cell signaling** |  |  |  |
| EF HAND CALCIUM BINDING PROTEIN 2 | 1.94 | 1418881_AT | NM_054095 |
| A KINASE (PRKA) ANCHOR PROTEIN 6 | 1.81 | 1440859_AT | NM_198111 |
|  |  |  |  |
| **Cellular metabolism/Enzymes** |  |  |  |
| MANNOSIDASE 1, ALPHA | 1.67 | 1417110_AT | NM_008548 |
| TORSIN FAMILY 1, MEMBER B | 1.62 | 1417820_AT | NM_133673 |
| PYRROLINE-5-CARBOXYLATE REDUCTASE 1 | 1.61 | 1424556_AT | NM_144795 |
| EPM2A (LAFORIN) INTERACTING PROTEIN 1 | 1.60 | 1454835_AT | NM_175266 |
| DIPEPTIDYLPEPTIDASE 7 | 1.51 | 1435680_A_AT | NM_031843 |
|  |  |  |  |
| **Cell proliferation** |  |  |  |
| TRANSFORMING, ACIDIC COILED-COIL CONTAINING PROTEIN 2 | 1.53 | 1425745_A_AT | NM_206856, NM_001004468, NM_021314 |
