# Supplementary material for: Alterations of Pancreatic Islet Structure, Metabolism and Gene Expression in Diet-Induced Obese C57BL/6J Mice
Source: PLoS One. 2014 Feb 5;9(2):e86815. doi: 10.1371/journal.pone.0086815 (PMC3914796; doi:10.1371/journal.pone.0086815)
Supplement: Table S2 — The list of genes down-regulated in HF islets compared with NC islets in the microarray analysis using a fold change cutoff of ≥1.5 and a false discovery rate of ≥0.13%. (DOC) [file pone.0086815.s002.doc]

Table S2

|  | Fold change | Affimetrix ID | REFSEQ_mRNA |
| --- | --- | --- | --- |
| **EXTRACELLULAR MATRIX** |  |  |  |
| COLLAGEN, TYPE I, ALPHA 1 | 0.09 | 1423669_AT | NM_007742 |
| OSTEOGLYCIN | 0.09, 0.26 | 1419662_AT 1419663_AT | NM_008760 |
| CYSTEINE-RICH SECRETORY PROTEIN LCCL DOMAIN CONTAINING 2 | 0.10, 0.12, 0.24 | 1434758_at1437056_x_at, 1460458_at | NM_030209 |
| VON WILLEBRAND FACTOR HOMOLOG | 0.12 | 1435386_at | NM_011708 |
| collagen, type XIV, alpha 1 | 0.15, 0.32 | 1427168_a_at, 1428455_at | NM_181277 |
| FIBROMODULIN | 0.15 | 1456084_x_at | NM_021355 |
| FIBULIN 2 | 0.16 | 1423407_a_at | NM_007992 |
| ELASTIN | 0.17 | 1420855_at | NM_007925 |
| COLLAGEN, TYPE I, ALPHA 2 | 0.17, 0.20 | 1450857_a_at, 1423110_at | NM_007743 |
| SPARC RELATED MODULAR CALCIUM BINDING 2 | 0.17 | 1415935_at | NM_022315 |
| LUMICAN | 0.19 | 1423607_at | NM_008524 |
| DECORIN | 0.19 | 1449368_at | NM_007833 |
| DERMATOPONTIN | 0.22 | 1418511_at | NM_019759 |
| TRANSGLUTAMINASE 2, C POLYPEPTIDE | 0.22, 0.27, 0.27, 0.28 | 1437277_x_at, 1417500_a_at, 1433428_x_at, 1455900_x_at | NM_009373 |
| FIBRILLIN 1 | 0.23 | 1460208_at | NM_007993 |
| PLEIOTROPHIN | 0.23 | 1448254_at | NM_008973 |
| collagen, type III, alpha 1 | 0.23, 0.25 | 1427883_a_at, 1427884_at | NM_009930 |
| PROLINE ARGININE-RICH END LEUCINE-RICH REPEAT | 0.23 | 1416321_s_at | NM_054077 |
| LECTIN, GALACTOSE BINDING, SOLUBLE 1 | 0.24, 0.30 | 1419573_a_at, 1455439_a_at | NM_008495 |
| BIGLYCAN | 0.25, 0.29, 0.43 | 1437889_x_at, 1448323_a_at, 1416405_at | NM_007542 |
| LYSYL OXIDASE-LIKE 1 | 0.26 | 1451978_at | NM_010729 |
| collagen, type VI, alpha 1 | 0.27 | 1448590_at | NM_009933 |
| MATRIX METALLOPEPTIDASE 2 | 0.27 | 1416136_at | NM_008610 |
| FIBULIN 1 | 0.28 | 1451119_a_at | NM_010180 |
| COLLAGEN, TYPE V, ALPHA 2 | 0.28 | 1422437_at | NM_007737 |
| coiled-coil domain containing 80 | 0.28 | 1424186_at | NM_026439 |
| ABI gene family, member 3 (NESH) binding protein | 0.29 | 1427054_s_at | NM_001014399 |
| HTRA SERINE PEPTIDASE 1 | 0.29 | 1416749_at | NM_019564 |
| CARTILAGE INTERMEDIATE LAYER PROTEIN, NUCLEOTIDE PYROPHOSPHOHYDROLASE | 0.29 | 1457296_at | NM_173385 |
| PERIOSTIN, OSTEOBLAST SPECIFIC FACTOR | 0.31 | 1423606_at | NM_015784 |
| TRANSFERRIN | 0.31 | 1425546_a_at | NM_133977 |
| FIBULIN 5 | 0.33 | 1416164_at | NM_011812 |
| EGF-like, fibronectin type III and laminin G domains | 0.33 | 1434647_at | NM_178748 |
| collagen, type VI, alpha 2 | 0.34, 0.45 | 1452250_a_at, 1426947_x_at | NM_146007 |
| TISSUE INHIBITOR OF METALLOPROTEINASE 2 | 0.35 | 1454677_at | NM_011594 |
| SPONDIN 1, (F-SPONDIN) EXTRACELLULAR MATRIX PROTEIN | 0.36 | 1451342_at | NM_145584 |
| extracellular matrix protein 2, female organ and adipocyte specific | 0.38 | 1440096_at | NM_001012324 |
| ANNEXIN A2 | 0.42 | 1419091_a_at | NM_007585 |
| COLLAGEN, TYPE IV, ALPHA 5 | 0.42 | 1425476_at | NM_007736 |
| collagen, type XV, alpha 1 | 0.43 | 1448755_at | NM_009928 |
| SERINE (OR CYSTEINE) PEPTIDASE INHIBITOR, CLADE H, MEMBER 1 | 0.43, 0.43 | 1450843_a_at, 1456733_x_at | NM_009825 |
| SPARC-LIKE 1 (MAST9, HEVIN) | 0.45 | 1416114_at | NM_010097 |
| NETRIN 1 | 0.45 | 1454974_at | NM_008744 |
| OLFACTOMEDIN-LIKE 2B | 0.46 | 1423915_at | NM_177068 |
| ASPORIN | 0.47 | 1448421_s_at | NM_025711 |
| VITRONECTIN | 0.48 | 1420484_a_at | NM_011707 |
| TRANSFORMING GROWTH FACTOR, BETA INDUCED | 0.49, 0.50 | 1415871_at 1456250_x_at | NM_009369 |
| ECTONUCLEOSIDE TRIPHOSPHATE DIPHOSPHOHYDROLASE 2 | 0.49 | 1418259_a_at | NM_009849 |
| MATRIX METALLOPEPTIDASE 14 (MEMBRANE-INSERTED) | 0.49 | 1448383_at | NM_008608 |
| SECRETED ACIDIC CYSTEINE RICH GLYCOPROTEIN | 0.50 | 1416589_at | NM_009242 |
| SERINE (OR CYSTEINE) PEPTIDASE INHIBITOR, CLADE E, MEMBER 2 | 0.50 | 1416666_at | NM_009255 |
|  |  |  |  |
| **IMMUNE RESPONSE/INFLMMATION** |  |  |  |
| immunoglobulin lambda chain complex | 0.03 | 1428719_at | XM_888208, XM_903342 |
| IMMUNOGLOBULIN LAMBDA CHAIN, VARIABLE 1 | 0.07, 0.17 | 1428720_s_at, 1430523_s_at | XM_888225 |
| IMMUNOGLOBULIN HEAVY CHAIN (J558 FAMILY) | 0.10 | 1421653_a_at | NM_001024700 |
| IMMUNOGLOBULIN JOINING CHAIN | 0.16 | 1424305_at | NM_152839 |
| CHEMOKINE (C-C MOTIF) LIGAND 21B | 0.16 | 1419426_s_at | NM_023052, NM_011335, NM_011124 |
| THYMUS CELL ANTIGEN 1, THETA | 0.16 | 1423135_at | NM_009382 |
| IMMUNOGLOBULIN HEAVY CHAIN 6 (HEAVY CHAIN OF IGM) | 0.18 | 1427351_s_at | XM_904359, XM_177464 |
| COMPLEMENT COMPONENT 4B (CHILDO BLOOD GROUP) | 0.18 | 1418021_at | NM_009780 |
| MEMBRANE-SPANNING 4-DOMAINS, SUBFAMILY A, MEMBER 4B | 0.20 | 1423467_at | NM_021718 |
| MYELIN AND LYMPHOCYTE PROTEIN, T-CELL DIFFERENTIATION PROTEIN | 0.23 | 1417275_at | NM_010762 |
| CD83 ANTIGEN | 0.23 | 1416111_at | NM_009856 |
| CD14 ANTIGEN | 0.24 | 1417268_at | NM_009841 |
| complement component 1, s subcomponent | 0.29 | 1424041_s_at | NM_144938 |
| CD53 ANTIGEN | 0.29 | 1448617_at | NM_007651 |
| PROTEIN C RECEPTOR, ENDOTHELIAL | 0.30 | 1420664_s_at | NM_011171 |
| COMPLEMENT COMPONENT FACTOR H | 0.32, 0.37 | 1450876_at1423153_x_at | NM_001029977, NM_001025575, NM_009888 |
| GUANYLATE NUCLEOTIDE BINDING PROTEIN 2 | 0.33 | 1418240_at | NM_010260 |
| FC RECEPTOR, IGG, LOW AFFINITY IIB | 0.34,0.37, 0.47 | 1435477_s_at, 1451941_a_at, 1455332_x_at | NM_010187 |
| AVIAN MUSCULOAPONEUROTIC FIBROSARCOMA (V-MAF) AS42 ONCOGENE HOMOLOG | 0.35 | 1456060_at | NM_001025577 |
| PROTEIN TYROSINE PHOSPHATASE, RECEPTOR TYPE, C | 0.36 | 1422124_a_at | NM_011210 |
| CHEMOKINE (C-X-C MOTIF) LIGAND 12 | 0.38, 0.43 | 1448823_at1417574_at | NM_001012477, NM_021704, NM_013655 |
| RHO, GDP DISSOCIATION INHIBITOR (GDI) BETA | 0.38 | 1426454_at | NM_007486 |
| histocompatibility 2, Q region locus 1; | 0.38 | 1431008_at | NM_207648 |
| MARCKS-LIKE 1 | 0.39 | 1437226_x_at | NM_010807 |
| RING FINGER PROTEIN 125 | 0.39 | 1429399_at | NM_026301 |
| MYELOID CELL NUCLEAR DIFFERENTIATION ANTIGEN | 0.39 | 1452348_s_at | NM_008329, NM_001033450, NM_172648 |
| EARLY B-CELL FACTOR 1 | 0.42 | 1448293_at | NM_007897 |
| INTERLEUKIN 2 RECEPTOR, GAMMA CHAIN | 0.42, 0.49 | 1416295_a_at, 1416296_at | NM_013563 |
| CD52 ANTIGEN | 0.42 | 1460218_at | NM_013706 |
| lysozyme 2 | 0.44 | 1423547_at | NM_017372 |
| CASPASE RECRUITMENT DOMAIN FAMILY, MEMBER 10 | 0.46 | 1449491_at | NM_130859 |
| INTERFERON INDUCED TRANSMEMBRANE PROTEIN 3 | 0.47 | 1423754_at | NM_025378 |
| HISTOCOMPATIBILITY 2, CLASS II ANTIGEN A, ALPHA | 0.47 | 1443783_x_at | NM_010378 |
| SERINE (OR CYSTEINE) PEPTIDASE INHIBITOR, CLADE G, MEMBER 1 | 0.48 | 1416625_at | NM_009776 |
| lysozyme 1 | 0.48, 0.50 | 1436996_x_at, 1439426_x_at | NM_013590 |
| fermitin family homolog 3 | 0.48 | 1456014_s_at | NM_153795 |
| LYMPHOCYTE CYTOSOLIC PROTEIN 1 | 0.49 | 1415983_at | NM_008879 |
| TUBULOINTERSTITIAL NEPHRITIS ANTIGEN-LIKE | 0.50 | 1417109_at | NM_023476 |
| FIBRINOGEN-LIKE PROTEIN 2 | 0.47 | 1421855_at | NM_008013 |
|  |  |  |  |
| **CYTOSKELETON/MUSCLE FIBER** |  |  |  |
| tubulin polymerization-promoting protein family member 3 | 0.19 | 1416713_at | NM_026481 |
| VIMENTIN | 0.25, 0.27, 0.29 | 1456292_a_at, 1450641_at 1438118_x_at | NM_011701 |
| CRYSTALLIN, ALPHA B | 0.28, 0.30 | 1416455_a_at, 1434369_a_at | NM_009964 |
| MYOSIN, LIGHT POLYPEPTIDE 9, REGULATORY | 0.30 | 1452670_at | NM_172118 |
| CORONIN, ACTIN BINDING PROTEIN 1A | 0.30, 0.38 | 1416246_a_at, 1455269_a_at | NM_009898 |
| PDZ AND LIM DOMAIN 1 (ELFIN) | 0.31 | 1416554_at | NM_016861 |
| AHNAK NUCLEOPROTEIN (DESMOYOKIN) | 0.33 | 1452217_at | NM_175108, NM_009643 |
| vestigial like 3 | 0.35 | 1453593_at | XM_283372 |
| ACTIN, ALPHA 2, SMOOTH MUSCLE, AORTA | 0.37 | 1416454_s_at | NM_007392 |
| CAPPING PROTEIN (ACTIN FILAMENT), GELSOLIN-LIKE | 0.38 | 1450355_a_at | NM_007599 |
| TRANSGELIN | 0.39 | 1423505_at | NM_011526 |
| FILAMIN, ALPHA | 0.40 | 1426677_at | NM_010227 |
| MYOSIN, HEAVY POLYPEPTIDE 11, SMOOTH MUSCLE | 0.42 | 1448962_at | NM_013607 |
| MYOSIN, LIGHT POLYPEPTIDE KINASE | 0.45 | 1425505_at1425506_at | NM_139300 |
| GELSOLIN | 0.47, 0.49 | 1415812_at 1456312_x_at | NM_146120 |
| FYVE, RHOGEF AND PH DOMAIN CONTAINING 5 | 0.47 | 1460578_at | NM_172731 |
| TROPOMYOSIN 2, BETA | 0.47 | 1419738_a_at | NM_009416 |
|  |  |  |  |
| **LIPIDS** |  |  |  |
| FATTY ACID BINDING PROTEIN 7, BRAIN | 0.13 | 1450779_at | NM_021272 |
| ACYL-COA THIOESTERASE 7 | 0.19 | 1417094_at | NM_133348 |
| RETINOL BINDING PROTEIN 1, CELLULAR | 0.22 | 1448754_at | NM_011254 |
| STEAROYL-COENZYME A DESATURASE 1 | 0.25 | 1415964_at | NM_009127 |
| APOLIPOPROTEIN D | 0.27 | 1416371_at | NM_007470 |
| ANNEXIN A1 | 0.28 | 1448213_at | NM_010730 |
| PROSTAGLANDIN-ENDOPEROXIDE SYNTHASE 1 | 0.41 | 1436448_a_at | NM_008969 |
| LEUCINE RICH REPEAT CONTAINING 8 FAMILY, MEMBER C | 0.42 | 1423614_at | NM_133897 |
| PHOSPHOLIPID TRANSFER PROTEIN | 0.43 | 1417963_at | NM_011125 |
| Alkylglycerol monooxygenase | 0.46 | 1434191_at | NM_178767 |
| SERUM DEPRIVATION RESPONSE | 0.47 | 1443832_s_at | NM_138741 |
| phospholipid scramblase 4 | 0.47 | 1433626_at | NM_178711 |
| FATTY ACID BINDING PROTEIN 4, ADIPOCYTE | 0.48 | 1417023_a_at | NM_024406 |
| CAVEOLIN, CAVEOLAE PROTEIN 1 | 0.49 | 1449145_a_at | NM_007616 |
| sphingosine-1-phosphate receptor 1 | 0.49 | 1423571_at | NM_007901 |
| FATTY ACID BINDING PROTEIN 4, ADIPOCYTE | 0.50 | 1451263_a_at | NM_024406 |
|  |  |  |  |
| **MEMBRANE RECEPTORS/MEMBRANE TRANSPORTERS** |  |  |  |
| POTASSIUM VOLTAGE-GATED CHANNEL, SHAKER-RELATED SUBFAMILY, MEMBER 1 | 0.10, 0.18 | 1455785_at1437230_at | NM_010595 |
| G PROTEIN-COUPLED RECEPTOR 126 | 0.15 | 1437409_s_at | NM_001002268 |
| SODIUM CHANNEL, VOLTAGE-GATED, TYPE VII, ALPHA | 0.14, 0.20 | 1436043_at1436044_at | NM_009135 |
| GLIAL CELL LINE DERIVED NEUROTROPHIC FACTOR FAMILY RECEPTOR ALPHA 2 | 0.17 | 1433716_x_at | NM_008115 |
| GLYCOPROTEIN M6B | 0.18 | 1423091_a_at | NM_023122 |
| PURINERGIC RECEPTOR P2Y, G-PROTEIN COUPLED 10 | 0.17 | 1452815_at | NM_172435 |
| MEMBRANE-SPANNING 4-DOMAINS, SUBFAMILY A, MEMBER 4D | 0.20 | 1418990_at | NM_025658 |
| SOLUTE CARRIER FAMILY 2 (FACILITATED GLUCOSE TRANSPORTER), MEMBER 3 | 0.21 | 1455898_x_at | NM_011401 |
| ATP-BINDING CASSETTE, SUB-FAMILY A (ABC1), MEMBER 8A | 0.21 | 1427371_at | NM_153145 |
| MYELIN BASIC PROTEIN | 0.26, 0.28, 0.37 | 1419646_a_at, 1433532_a_at, 1456228_x_at | NM_001025258, NM_010777, NM_001025251, NM_001025259, NM_001025256, NM_001025255, NM_001025245, NM_001025254 |
| ANTHRAX TOXIN RECEPTOR 2 | 0.28 | 1426708_at | NM_133738 |
| PROTEIN C RECEPTOR, ENDOTHELIAL | 0.30 | 1420664_s_at | NM_011171 |
| PROTEOLIPID PROTEIN (MYELIN) 1 | 0.30, 0.38 | 1425468_at, 1451718_at | NM_011123 |
| LEPTIN RECEPTOR | 0.32 | 1456156_at | NM_010704, NM_146146 |
| apelin receptor | 0.34 | 1438651_a_at | NM_011784 |
| POTASSIUM VOLTAGE-GATED CHANNEL, ISK-RELATED SUBFAMILY, GENE 4 | 0.34 | 1418156_at | NM_021342 |
| CHEMOKINE ORPHAN RECEPTOR 1 | 0.35 | 1417625_s_at | NM_007722 |
| SOLUTE CARRIER ORGANIC ANION TRANSPORTER FAMILY, MEMBER 2B1 | 0.36 | 1433933_s_at | NM_175316 |
| MANNOSE RECEPTOR, C TYPE 1 | 0.37 | 1450430_at | NM_008625 |
| ENDOTHELIAL DIFFERENTIATION, SPHINGOLIPID G-PROTEIN-COUPLED RECEPTOR, 3 | 0.38 | 1438658_a_at | NM_010101 |
| G PROTEIN-COUPLED RECEPTOR 146 | 0.38, 0.40 | 1451060_at1454685_at | NM_030258, NM_001038703 |
| ANTHRAX TOXIN RECEPTOR 1 | 0.39 | 1451446_at | NM_054041 |
| INTEGRAL MEMBRANE PROTEIN 2A | 0.39 | 1423608_at | NM_008409 |
| peripheral myelin protein 22 | 0.40 | 1417133_at | NM_008885 |
| PROSTAGLANDIN F2 RECEPTOR NEGATIVE REGULATOR | 0.40 | 1434891_at | NM_011197 |
| MEMBRANE-SPANNING 4-DOMAINS, SUBFAMILY A, MEMBER 6B | 0.41 | 1418826_at | NM_027209 |
| ALS2 C-TERMINAL LIKE | 0.42 | 1433769_at | NM_146228 |
| ATPASE, NA+/K+ TRANSPORTING, ALPHA 2 POLYPEPTIDE | 0.43 | 1452308_a_at | NM_178405 |
| SOMATOSTATIN RECEPTOR 2 | 0.42 | 1422256_at | NM_009217 |
| V-ERB-B2 ERYTHROBLASTIC LEUKEMIA VIRAL ONCOGENE HOMOLOG 3 (AVIAN) | 0.45 | 1434606_at | NM_010153 |
| HIG1 DOMAIN FAMILY, MEMBER 1A | 0.45 | 1416480_a_at | NM_019814 |
| ONCOSTATIN M RECEPTOR | 0.47 | 1418674_at | NM_011019 |
| ATP-BINDING CASSETTE, SUB-FAMILY G (WHITE), MEMBER 2 | 0.48 | 1422906_at | NM_011920 |
| LIPOMA HMGIC FUSION PARTNER | 0.48 | 1433776_at | NM_175386 |
| LOW DENSITY LIPOPROTEIN RECEPTOR-RELATED PROTEIN 1 | 0.50 | 1448655_at | NM_008512 |
| SYNDECAN 1 | 0.50 | 1437279_x_at | NM_011519 |
|  |  |  |  |
| **ADHESION/CELL JUNCTION** |  |  |  |
| CADHERIN 19, TYPE 2 | 0.09 | 1457373_at | NM_001081386 |
| CADHERIN 11 | 0.16 | 1450757_at | NM_009866 |
| VASCULAR CELL ADHESION MOLECULE 1 | 0.20 | 1448162_at | NM_011693 |
| EPITHELIAL MEMBRANE PROTEIN 1 | 0.27 | 1416529_at | NM_010128 |
| CD9 ANTIGEN | 0.33 | 1416066_at | NM_007657 |
| PROTOCADHERIN 8 | 0.38 | 1417051_at | NM_021543 |
| CD302 ANTIGEN | 0.39 | 1448919_at | NM_025422 |
| TENSIN 1 | 0.45 | 1428650_at | NM_027884 |
|  |  |  |  |
| **PEPTIDE/HORMONE/CYTOKINES** |  |  |  |
| GASTRIN RELEASING PEPTIDE | 0.06 | 1424525_at | NM_175012 |
| VASOACTIVE INTESTINAL POLYPEPTIDE | 0.06 | 1428664_at | NM_011702 |
| TACHYKININ 1 | 0.11 | 1416783_at | NM_009311 |
| PREPROENKEPHALIN 1 | 0.18 | 1427038_at | NM_001002927 |
| GROWTH DIFFERENTIATION FACTOR 10 | 0.19 | 1424007_at | NM_145741 |
| SECRETED FRIZZLED-RELATED SEQUENCE PROTEIN 1 | 0.19 | 1428136_at | NM_013834 |
| neuropeptide Y | 0.20 | 1419127_at | NM_023456 |
| DIPEPTIDASE 1 (RENAL) | 0.25 | 1435943_at | NM_007876 |
| SCLEROSTIN DOMAIN CONTAINING 1 | 0.39 | 1449340_at | NM_025312 |
| RETINOIC ACID RECEPTOR RESPONDER (TAZAROTENE INDUCED) 2 | 0.42, 0.47 | 1428538_s_at, 1437902_s_at | NM_027852 |
| PLACENTA SPECIFIC 9 | 0.43, 0.46 | 1458659_at1452590_a_at | NM_207229 |
| PROTEIN S (ALPHA) | 0.46 | 1426246_at | NM_011173 |
| OLFACTOMEDIN 3 | 0.48 | 1425898_x_at | NM_153458, NM_153157 |
|  |  |  |  |
| **CELL METABOLISM/GROWTH/APOPTOSIS/TRANSCRIPTION** |  |  |  |
| SECRETED FRIZZLED-RELATED SEQUENCE PROTEIN 1 | 0.09 | 1460187_at | NM_013834 |
| FLAVIN CONTAINING MONOOXYGENASE 2 | 0.15, 0.35 | 1435459_at1453435_a_at | NM_018881 |
| INSULIN-LIKE GROWTH FACTOR BINDING PROTEIN 4 | 0.16, 0.19, 0.22, 0.40, 0.48 | 1423756_s_at, 1423757_x_at, 1437405_a_at, 1437406_x_at, 1421992_a_at | NM_010517 |
| HYDROXYSTEROID 11-BETA DEHYDROGENASE 1 | 0.22 | 1449038_at | NM_008288 |
| GULP, ENGULFMENT ADAPTOR PTB DOMAIN CONTAINING 1 | 0.26 | 1434423_at | NM_027506, NM_028450 |
| SERINE/THREONINE KINASE 17B (APOPTOSIS-INDUCING) | 0.29, 0.34 | 1450997_at1423452_at | NM_133810 |
| SELENIUM BINDING PROTEIN 1 | 0.30 | 1417580_s_at | NM_009150 |
| PLECKSTRIN HOMOLOGY, SEC7 AND COILED-COIL DOMAINS, BINDING PROTEIN | 0.30 | 1451206_s_at | NM_139200 |
| CERULOPLASMIN | 0.14, 0.31, 0.41, 0.52 | 1417495_x_at, 1417494_a_at, 1417495_x_at, 1417496_at1448734_at1448735_at | NM_007752 |
| INDOLETHYLAMINE N-METHYLTRANSFERASE | 0.33 | 1418697_at | NM_009349 |
| S100 CALCIUM BINDING PROTEIN A6 (CALCYCLIN) | 0.34 | 1421375_a_at | NM_011313 |
| FOLLISTATIN-LIKE 1 | 0.31, 0.38 | 1416221_at1448259_at | NM_008047 |
| BRAIN ABUNDANT, MEMBRANE ATTACHED SIGNAL PROTEIN 1 | 0.36 | 1428572_at | NM_027395 |
| SLIT HOMOLOG 2 (DROSOPHILA) | 0.37 | 1424659_at | NM_178804 |
| INSULIN-LIKE GROWTH FACTOR BINDING PROTEIN 5 | 0.38 | 1452114_s_at | NM_010518 |
| MICROSOMAL GLUTATHIONE S-TRANSFERASE 1 | 0.40 | 1415897_a_at | NM_019946 |
| JUN DIMERIZATION PROTEIN 2 | 0.40 | 1450350_a_at | NM_030887 |
| AMINE OXIDASE, COPPER CONTAINING 3 | 0.42 | 1449396_at | NM_009675 |
| PHOSPHODIESTERASE 2A, CGMP-STIMULATED | 0.42 | 1452202_at | NM_001008548 |
| LACTATE DEHYDROGENASE A | 0.43 | 1419737_a_at | NM_010699 |
| FRIEND LEUKEMIA INTEGRATION 1 | 0.43 | 1433512_at | NM_008026 |
| NUCLEAR FACTOR I/B | 0.43, 0.43 | 1448288_at1427680_a_at | NM_008687 |
| KRUPPEL-LIKE FACTOR 4 (GUT) | 0.43 | 1417394_at | NM_010637 |
| NUCLEAR RECEPTOR SUBFAMILY 2, GROUP F, MEMBER 2 | 0.44 | 1416158_at | NM_183261, NM_009697 |
| CASPASE 12 | 0.44 | 1449297_at | NM_009808 |
| KRUPPEL-LIKE FACTOR 2 (LUNG) | 0.44 | 1448890_at | NM_008452 |
| PYRUVATE DEHYDROGENASE KINASE, ISOENZYME 4 | 0.45 | 1417273_at | NM_013743 |
| GULP, ENGULFMENT ADAPTOR PTB DOMAIN CONTAINING 1 | 0.45 | 1453771_at | NM_027506, NM_028450 |
| ORNITHINE AMINOTRANSFERASE | 0.45 | 1416452_at | NM_016978 |
| ZINC FINGER PROTEIN 521 | 0.47 | 1451332_at | NM_181326, NM_145492 |
| MESODERM SPECIFIC TRANSCRIPT | 0.47 | 1423294_at | NM_008590 |
| ZINC FINGER HOMEODOMAIN 4 | 0.47 | 1437556_at | NM_030708 |
| SCAVENGER RECEPTOR CLASS A, MEMBER 3 | 0.48 | 1427020_at | NM_172604 |
| IMMEDIATE EARLY RESPONSE 3 | 0.48 | 1419647_a_at | NM_133662 |
| GUANYLATE CYCLASE 1, SOLUBLE, ALPHA 3 | 0.48 | 1434141_at | NM_021896 |
| INTERFERON INDUCIBLE GTPASE 1 | 0.49 | 1419043_a_at | NM_021792 |
|  |  |  |  |
|  |  |  |  |
| **CELL SGINALING** |  |  |  |
| ADENYLATE CYCLASE 7 | 0.25, 0.49 | 1456307_s_at, 1450065_at | NM_001037724, NM_007406, NM_001037723 |
| PHOSPHOLIPASE C-LIKE PROTEIN | 0.35 | 1452398_at | NM_019588 |
| PROTEIN TYROSINE PHOSPHATASE, RECEPTOR TYPE, C | 0.36 | 1422124_a_at | NM_011210 |
| ZINC FINGER HOMEOBOX 1B | 0.39, 0.41 | 1456389_at1434298_at | NM_001033635, NM_015753 |
| RAB27B, MEMBER RAS ONCOGENE FAMILY | 0.49 | 1439610_at | NM_030554 |
| CAVEOLIN, CAVEOLAE PROTEIN 1 | 0.49 | 1449145_a_at | NM_007616 |
|  |  |  |  |
| **Others** |  |  |  |
| IMMUNOGLOBULIN SUPERFAMILY CONTAINING LEUCINE-RICH REPEAT | 0.25 | 1418450_at | NM_012043 |
| HtrA serine peptidase 3 | 0.25 | 1419292_at | NM_030127 |
| GENE MODEL 1186, (NCBI) | 0.26 | 1455530_at | XM_356604 |
| GLYCOPROTEIN M6B | 0.26 | 1425942_a_at | NM_023122 |
| CARBOXYPEPTIDASE X 2 (M14 FAMILY) | 0.28 | 1460248_at | NM_018867 |
| family with sequence similarity 198, member B | 0.30 | 1416805_at | NM_133187 |
| RIKEN CDNA 9130213B05 GENE | 0.33 | 1428891_at | NM_145562 |
| INTERFERON ACTIVATED GENE 205 | 0.36 | 1452349_x_at | NM_172648, NM_001033450 |
| RIKEN CDNA 3110001A13 GENE | 0.39 | 1416892_s_at | NM_025626 |
| RIKEN CDNA 3010015F07 GENE | 0.43 | 1420514_at | NM_138751, NM_175771 |
| EXPRESSED SEQUENCE AW551984 | 0.43 | 1433434_at | NM_178737 |
| epithelial stromal interaction 1 (breast) | 0.46 | 1452087_at | NM_178825, NM_029495 |
| IMMUNOGLOBIN SUPERFAMILY, MEMBER 21 | 0.46 | 1433950_at | NM_198610 |
| TRANSMEMBRANE 6 SUPERFAMILY MEMBER 1 | 0.46 | 1424443_at | NM_145375 |
| RIKEN CDNA 3110001A13 GENE | 0.47 | 1416893_at | NM_025626 |
